# PARP-dependent and NAT10-independent acetylation of N4-cytidine in RNA appears in UV-damaged chromatin

**DOI:** 10.1186/s13072-023-00501-x

**Published:** 2023-06-15

**Authors:** Alena Svobodová Kovaříková, Lenka Stixová, Aleš Kovařík, Eva Bártová

**Affiliations:** Department of Cell Biology and Epigenetics, Institute of Biophysics, Academy of Sciences of the Czech Republic, Královopolská 135, Brno, Czech Republic

**Keywords:** RNA acetylation, RNA methylation, NAT10, DNA repair, PARP

## Abstract

RNA modifications have been known for many years, but their function has not been fully elucidated yet. For instance, the regulatory role of acetylation on N4-cytidine (ac4C) in RNA can be explored not only in terms of RNA stability and mRNA translation but also in DNA repair. Here, we observe a high level of ac4C RNA at DNA lesions in interphase cells and irradiated cells in telophase. Ac4C RNA appears in the damaged genome from 2 to 45 min after microirradiation. However, RNA cytidine acetyltransferase NAT10 did not accumulate to damaged sites, and NAT10 depletion did not affect the pronounced recruitment of ac4C RNA to DNA lesions. This process was not dependent on the G1, S, and G2 cell cycle phases. In addition, we observed that the PARP inhibitor, olaparib, prevents the recruitment of ac4C RNA to damaged chromatin. Our data imply that the acetylation of N4-cytidine, especially in small RNAs, has an important role in mediating DNA damage repair. Ac4C RNA likely causes de-condensation of chromatin in the vicinity of DNA lesions, making it accessible for other DNA repair factors involved in the DNA damage response. Alternatively, RNA modifications, including ac4C, could be direct markers of damaged RNAs.

**Supplementary Information:**

The online version contains supplementary material available at 10.1186/s13072-023-00501-x.

## Introduction

In general, acetylation is a well-described cellular mechanism that regulates gene expression, especially in relation to histones [1]. N4-acetylcytidine (ac4C), a highly conserved RNA nucleobase, also contributes to the regulation of mRNA stability and efficiency of translation [2]. Information about the function of ac4C RNA in the DNA repair process has not been published yet, but levels of histone acetylation are known to change when chromatin is damaged [3]. For instance, we recently observed HDAC1-dependent deacetylation of histone H3 lysine 9 (H3K9) in experimentally induced DNA lesions [4]. On the other hand, Meyer et al. [5] showed that H3K9 acetylation prevents H3K9 methylation, thereby inhibiting H3K9me2/3-dependent DNA repair processes. Dhar et al. [6] observed that histone H4 terminal tails recruit proteins involved in DNA damage response (DDR), including 53BP1, an important factor of the non-homologous end joining (NHEJ) mechanism. This process is accompanied by changes in H4 acetylation. Among histones, a fundamental marker of double-strand breaks (DSBs) is the phosphorylation of histone H2AX (γH2AX). Also, H2A.Z exchange at DSB sites occurs in cooperation with the ATPase function of P400 [7, 8]. Data from these studies show that the histone code directly regulates DNA damage repair. Additionally, Ikura et al. [9] showed that poly (ADP-ribose) polymerase 1 (PARP-1) is required for the rapid exchange of H2AX on damaged chromatin. It is known that the PARP-1 protein is recruited dynamically to DNA lesions and that TIP60-mediated H2AX acetylation at lysine 5, but not γH2AX, is required for the ADP-ribosylation activity of PARP-1 at sites of genome damage [9].

When considering the function of acetylation processes as part of the DNA repair machinery, we also took into consideration the existence of N4-cytidine in RNA [10]. It is known that the acetylation of N4-cytidine in RNA occurs via the function of N-acetyltransferase, NAT10 [11–14]. The target of the NAT10 enzyme is preferentially rRNA and tRNA (tRNA-Ser and tRNA-Leu) [12, 13, 15]. Kudrin et al. [16] newly identified NOP58 as an ac4C-binding protein and, importantly, sirtuin 7 (SIRT7) as a specific ac4C deacetylase. Thus, NAT10 can be considered the essential "writer" of ac4C in distinct types of RNA. NOP58 represents an ac4C RNA "reader," while SIRT7 seems to be a highly specific ac4C RNA "eraser." Furthermore, ac4C is installed in mRNA at physiologically relevant levels and is essential for mRNA stability and translation. Importantly, Arago et al. [2] showed that ac4C is highly abundant in the human transcriptome, and NAT10 gene down-regulation reduced the level of ac4C in mRNA, thus affecting gene expression [2]. Furthermore, Arago et al. [17] showed that mRNA acetylation regulates the process of translation in a location-specific manner. Recently, it was reported that N-acetyltransferase 10 (NAT10) is responsible for ac4C modification in long noncoding RNAs (lncRNAs) [18].

Based on this observation, we addressed the question of how ac4C RNAs contribute to the DNA repair machinery and if the regulatory protein NAT10 contributes to the process of DNA damage repair. Our additional aim was to reveal in which DNA repair pathways ac4C RNA is involved. We were inspired by the fact that the level of other RNA modifications, including m^6^A and m^8^A, are significantly higher in UV-damaged chromatin, which is mainly recognized by the base excision repair (BER) or nucleotide excision repair (NER) mechanisms [19, 20]. Notably, a non-canonical m^6^A-mediated DNA repair pathway was also described, which is dependent on METTL3 and METTL14 enzymes or PARP1/2 proteins [21]. To these facts, we also studied whether ac4C RNA recruitment to DNA lesions is PARP-dependent. Based on our results, we summarize that in damaged chromatin, there is a high level of acetylated RNA, and we suggest that ac4C RNA could contribute to chromatin relaxation in the vicinity of DNA lesions. Alternatively, RNA modifications, including m^6^A, m^8^A, and ac4C, might be direct markers of damaged RNA.

## Results

### UVA and UVC-induced DNA lesions are recognized by ac4C RNA irrespective of cell cycle phase during interphase

Here, we were interested in whether ac4C RNA contributes to DNA repair mechanisms. Initially, by employing enzymatic treatment, we verified the specificity of our antibodies against ac4C in RNA. Using immunohistochemistry, we observed a significantly reduced level of ac4C RNA after RNase A treatment; this reduction was mainly in nucleoli, consisting of the fibrillar center (FC), the dense fibrillar component (DFC), and the granular component (GC) (Additional file 1: Fig. S1A, B). After RNase H1 treatment, the ac4C-related fluorescent intensity did not significantly change in interphase cell nuclei (Additional file 1: Fig. S1A, B). Similarly, DNase I did not alter the ac4C RNA level; however, DNA was degraded, as visualized by DAPI staining (Additional file 1: Fig. S1A, C). Also, analysis of total RNA by anti-ac4C, used in dot blots, documented a significantly reduced level of ac4C RNA in the RNase A-treated samples compared to untreated samples or those cells treated by RNase H1 or DNase I (Additional file 1: Fig. S1D). These dot blot results were verified by two antibodies against N4-acetylcytidine (#ab252215, Abcam and #A18806, Abclonal), and are consistent with further immunohistochemistry data (Additional file 1: Fig. S1A, B, D). Next, we induced chemical deacetylation using hydroxylamine [22] to verify the specificity of the two ac4C antibodies used. Dot blots showed that the level of ac4C was significantly reduced in hydroxylamine-treated samples when total RNA isolated from immortalized MEFs was analyzed (Additional file 1: Fig. S1E).

**Fig. 1:**
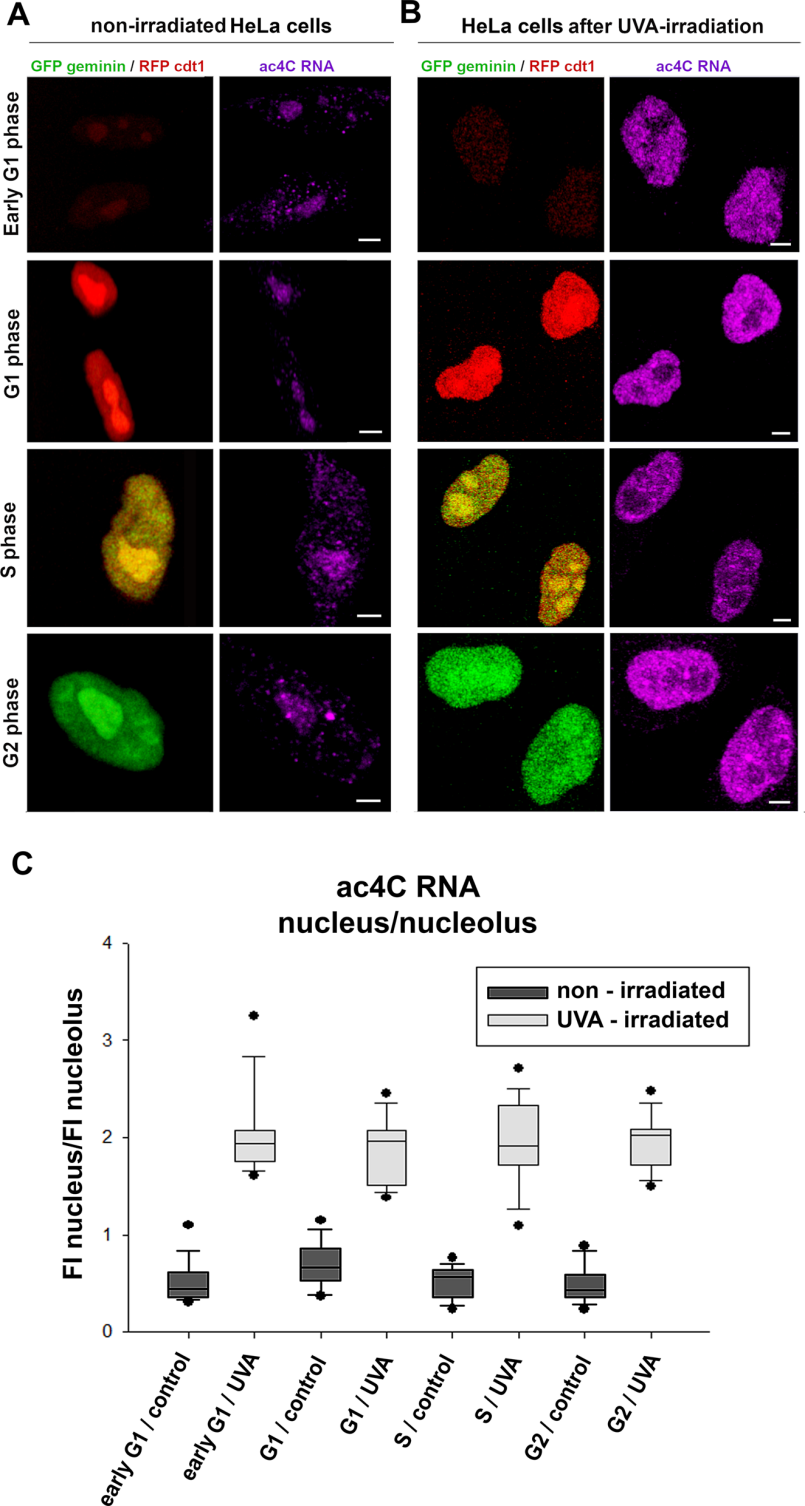
Increased levels of ac4C RNAs after UV irradiation are identical in the G1, S, and G2 phases of the cell cycle. The density of ac4C RNA was detected in **A** non-irradiated HeLa Fucci cells and **B** UVA-irradiated HeLa Fucci cells, stably expressing RFP-tagged cdt1 (red) in the G1 phase and GFP-tagged geminin (green) in the G2 phase of the cell cycle. The S phase is characterized by RFP-cdt1 and GFP-geminin positivity. Scale bars show 5 µm. **C** Quantification of the fluorescence intensity of ac4C RNA is shown in panels A and B

**Fig. 2:**
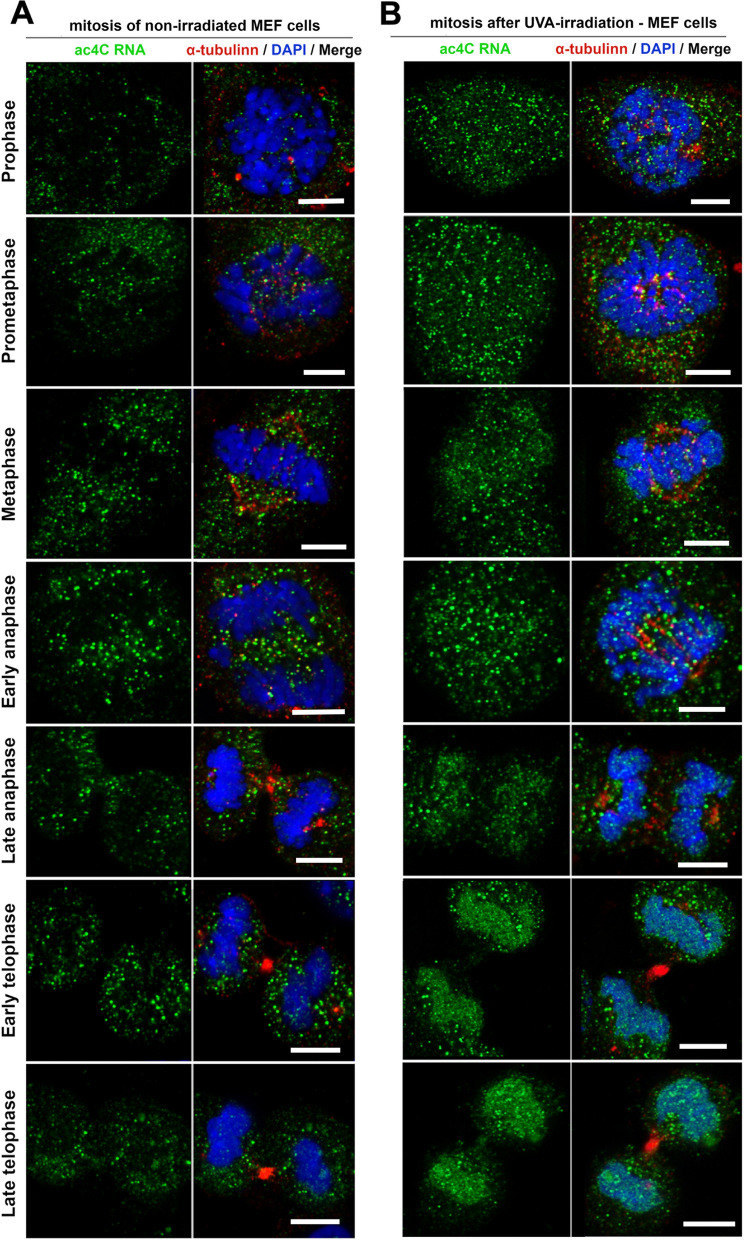
Increased levels of ac4C RNAs after UV-irradiation in early and late telophase of mitotic cells. The distribution profile of ac4C RNA (green fluorescence) was studied in mitotic cells of **A** non-irradiated MEF cells and **B** UV-irradiated MEFs. The following mitotic phases were distinguished: prophase, prometaphase, metaphase, early anaphase, late anaphase, early telophase, and late telophase. DNA was stained by DAPI (blue), and α-tubulin is shown in red fluorescence. Scale bars show 15 µm

As the next step, we used HeLa-Fucci cells highly expressing RFP-cdt1 in the G1 phase and GFP-tagged geminin in the G2 phase of the cell cycle. The S phase of the cell cycle was characterized by the expression of both RFP-cdt1 and GFP-tagged geminin [23]. In these cells, we studied ac4C RNA levels in non-irradiated cells and in whole cell populations irradiated by UVA light (Fig. 1A, B). In the G1, S, and G2 cell cycle phases, we observed an identical increase in ac4C RNA levels in UVA-irradiated cells compared to their non-irradiated counterparts (Fig. 1A–C). Also, we studied the ac4C RNA profile in mitotic cells, and we found that UVA irradiation caused an increase in ac4C RNA decorating mitotic chromosomes. In the early and late telophase of UVA-irradiated cells, we observed the highest density of ac4C RNA (Fig. 2A, B).

**Fig. 3:**
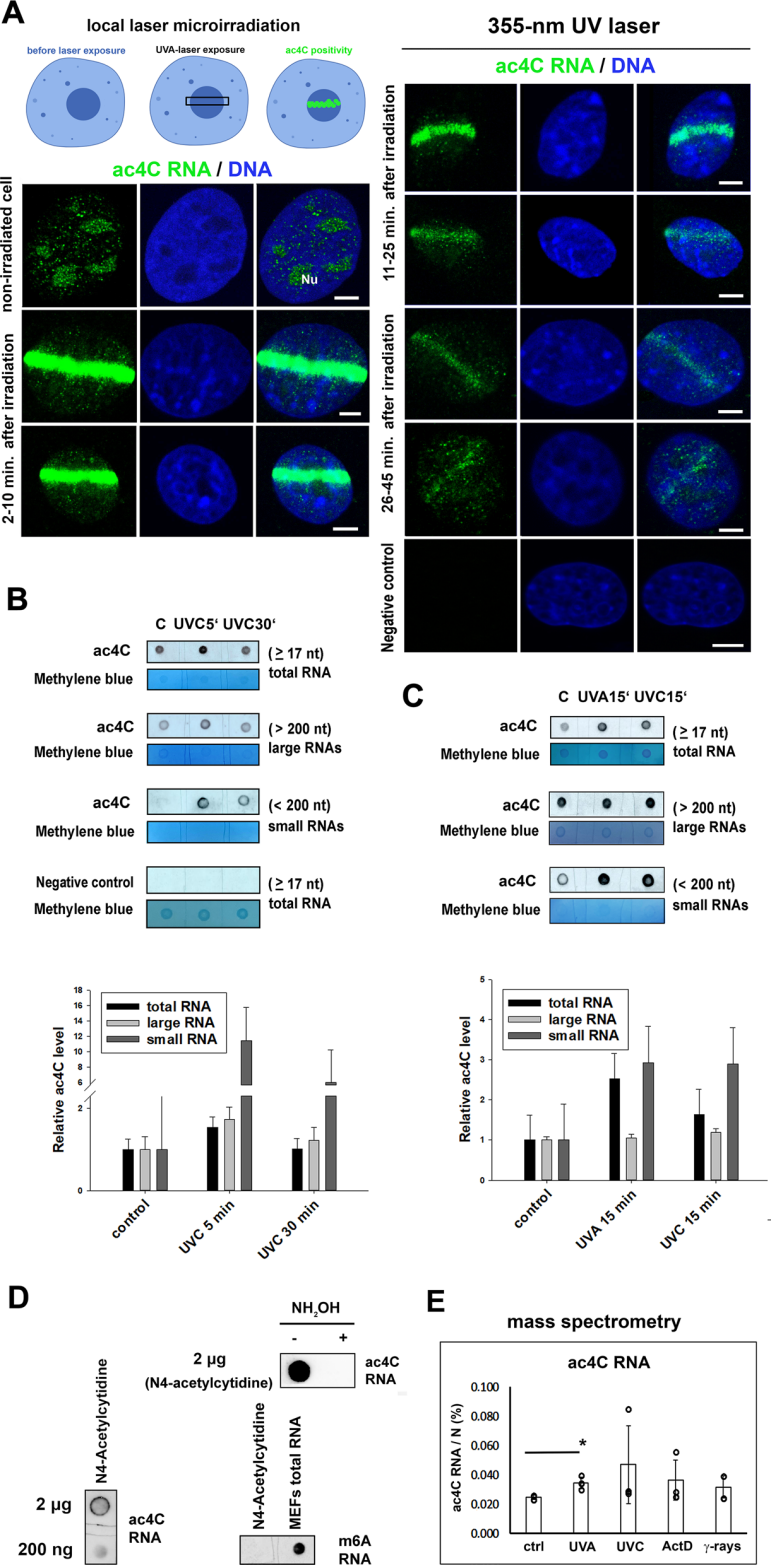
Recruitment of ac4C RNAs to UVA- and UVC-damaged chromatin. **A** Local microirradiation by 355-nm laser line showed that ac4C RNA recognizes UVA-microirradiated chromatin immediately after laser irradiation. In the later stages of DDR, 11–45 min post-irradiation, the ac4C RNA signal at DNA lesions was reduced. Scale bars are 5 µm. **B**, **C** Dot blot analysis of ac4C RNA documents levels of ac4C in total RNA isolated from non-irradiated, UVA-, and UVC-irradiated MEF cells. **B** shows the level of ac4C in RNAs (#ab252215, Abcam) studied 5 min and 30 min after UVC irradiation, and panel **C** documents the density of ac4C in RNAs (#ab252215, Abcam) analyzed 15 min after UVA and UVC irradiation. Negative controls (samples not incubated with the primary antibody) are shown. After fractionalization, it was observed that both large RNAs and small RNAs were notably acetylated on N4-cytidine when the cells were exposed to UVC light for 5 min, while 15 min post-irradiation, the highest level of ac4C was on small RNAs. Quantification of the density of dot spots is shown for both panels **B** and **C**. **D** Representative anti-ac4C dot blot (#ab252215, Abcam) performed on N4-acetylcytidine (NA05753, Biosynth). Chemical deacetylation was induced by hydroxylamine (50 mM, pH7, 65 °C, 1 h) [22]. The specificity of N4-acetylcytidine (NA05753, Biosynth) was verified by antibody against m^6^A in RNA (#202 111, SYSY Antibodies). **E** Mass spectrometry data on ac4C in total and large RNAs studied in control, non-treated cells, and cells exposed to UVA, UVC light, and γ-rays. Cells were also treated with actinomycin D (ActD). **E** shows the mean ± standard deviations (SD) for three biological replicates (n = 3). Asterisks (*) show p ≤ 0.05, calculated using the two-tailed Student's t-test, indicating statistically significant differences in the level of ac4C RNA

Application of the local laser microirradiation technique showed a high level of ac4C RNA in UVA-damaged chromatin immediately after genome injury. The ac4C RNA signal diminished 11–45 min post-irradiation (Fig. 3A). Dot blots confirmed an increased overall level of ac4C in total RNA of UVA-irradiated cells and cells exposed to UVC light (Fig. 3B, C). For these samples, we additionally performed RNA fractionalization into large and small RNAs (Fig. 3B, C, Additional file 1: Fig. S1F). In comparison to non-irradiated cells, dot blot analysis showed that both large and small RNAs were highly acetylated for up to 5 min after UVC irradiation, and small RNAs remained highly acetylated up to 30 min post-irradiation (Fig. 3B). In contrast to large RNAs, small RNAs were more frequently acetylated on N4-cytidine 15 min. after UVA and UVC irradiation when compared to non-irradiated counterparts (Fig. 3C). Furthermore, for data verification, we used N4-acetylcytidine (NA05753, Biosynth), which is a modified nucleoside and endogenous urinary nucleoside product of the degraded tRNA. The specificity of N4-acetylcytidine (NA05753, Biosynth) we verified by antibody against m^6^A RNA **(**#202 111, SYSY Antibodies) (Fig. 3D). Also, we validated the specificity of ac4C antibody (ab252215) by dot blot analysis that demonstrates concentration-dependent detection of ac4C in RNA (Fig. 3D). To this fact, the ac4C signal was significantly reduced by chemical ablation using hydroxylamine (NH_2_OH) (Fig. 3D).

An increase in ac4C RNA caused by the UV-irradiation we also confirmed by mass spectrometry. We analyzed ac4C levels in total and long RNAs isolated from HeLa cells exposed to UVA, UVC light, and gamma radiation using LC–MS. Mass spectrometry showed that, compared with non-irradiated control cells, UVA light increased the level of ac4C in RNA studied. This conclusion is based on a statistical analysis of mass spectrometry data (Fig. 3E, Additional file 2: Table S1).

**Fig. 4:**
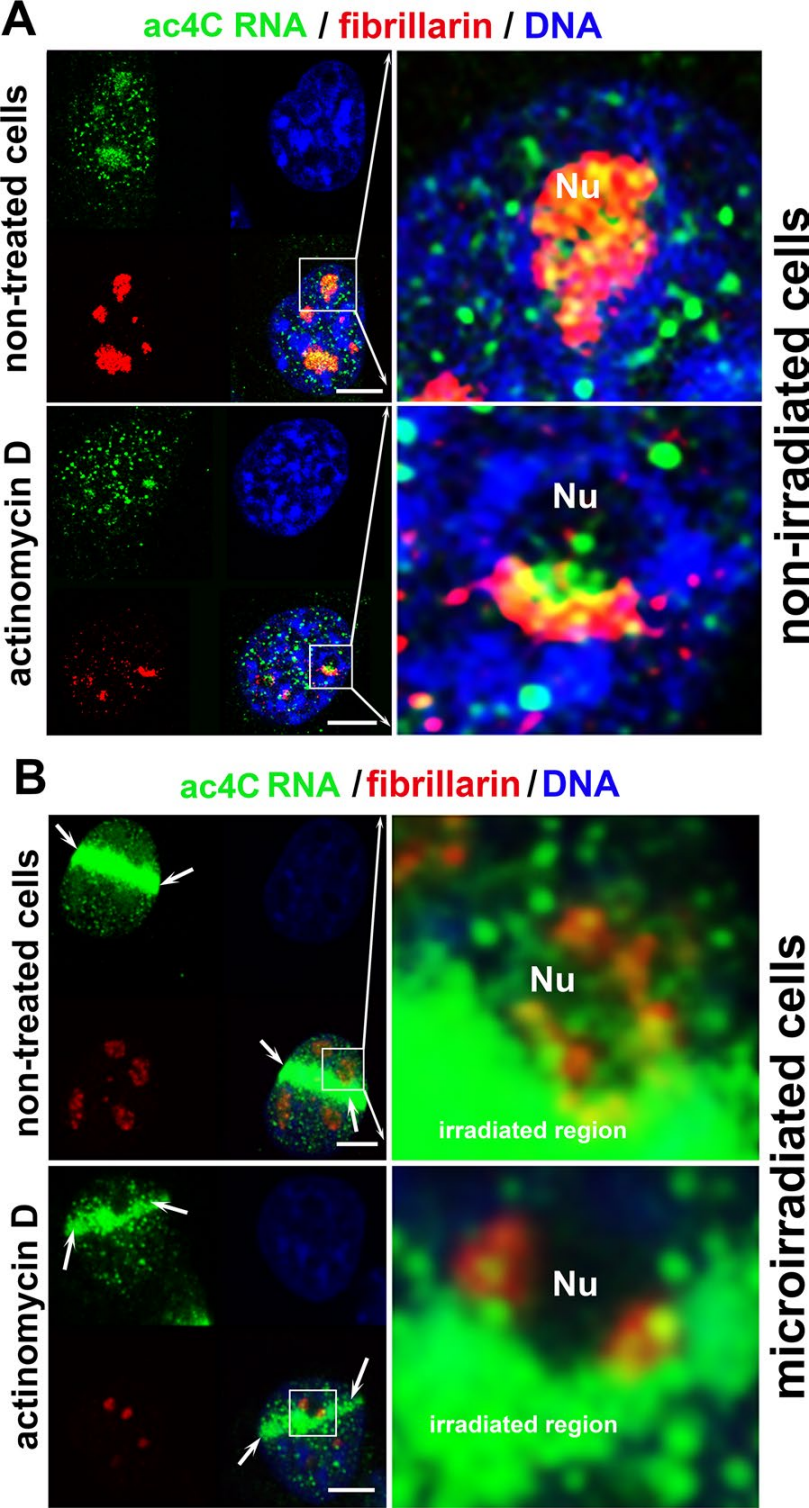
A high density of ac4C RNA is inside nucleoli on non-irradiated cells, and ac4C RNA colocalizes with fibrillarin. **A**, **B** Ac4C RNAs colocalize with fibrillarin-positive regions of nucleoli in non-irradiated cells. The same protein-RNA colocalization was observed in MEFs treated with actinomycin D, an inhibitor of RNA pol I. In these cells, actinomycin D treatment caused a crescent-like morphology of nucleoli, visualized by antibodies against fibrillarin. Analysis was also performed in UVA-microirradiated cells. Scale bars are 5 µm

In non-irradiated cell nuclei, we observed that ac4C RNA mainly occupies the specific nucleolar region (Fig. 3A). This phenomenon was additionally verified by dual immunolabelling showing in parallel ac4C RNA and fibrillarin, a well-known protein of the dense fibrillar component (DFC) of nucleoli (Fig. 4A, B). The fibrillarin-positive region of the nucleoli of non-irradiated cells was abundant on ac4C RNA, and the RNA polymerase I inhibitor actinomycin D induced crescent-like morphology of the nucleolar regions positive on both fibrillarin and ac4C RNA. These actinomycin D-induced structures precisely colocalized with ac4C RNAs in both non-irradiated and microirradiated cells (Fig. 4A, B). Moreover, mass spectrometry showed an equivalent level of ac4C RNA in control cells and cells treated with actinomycin D (Fig. 3E).

**Fig. 5:**
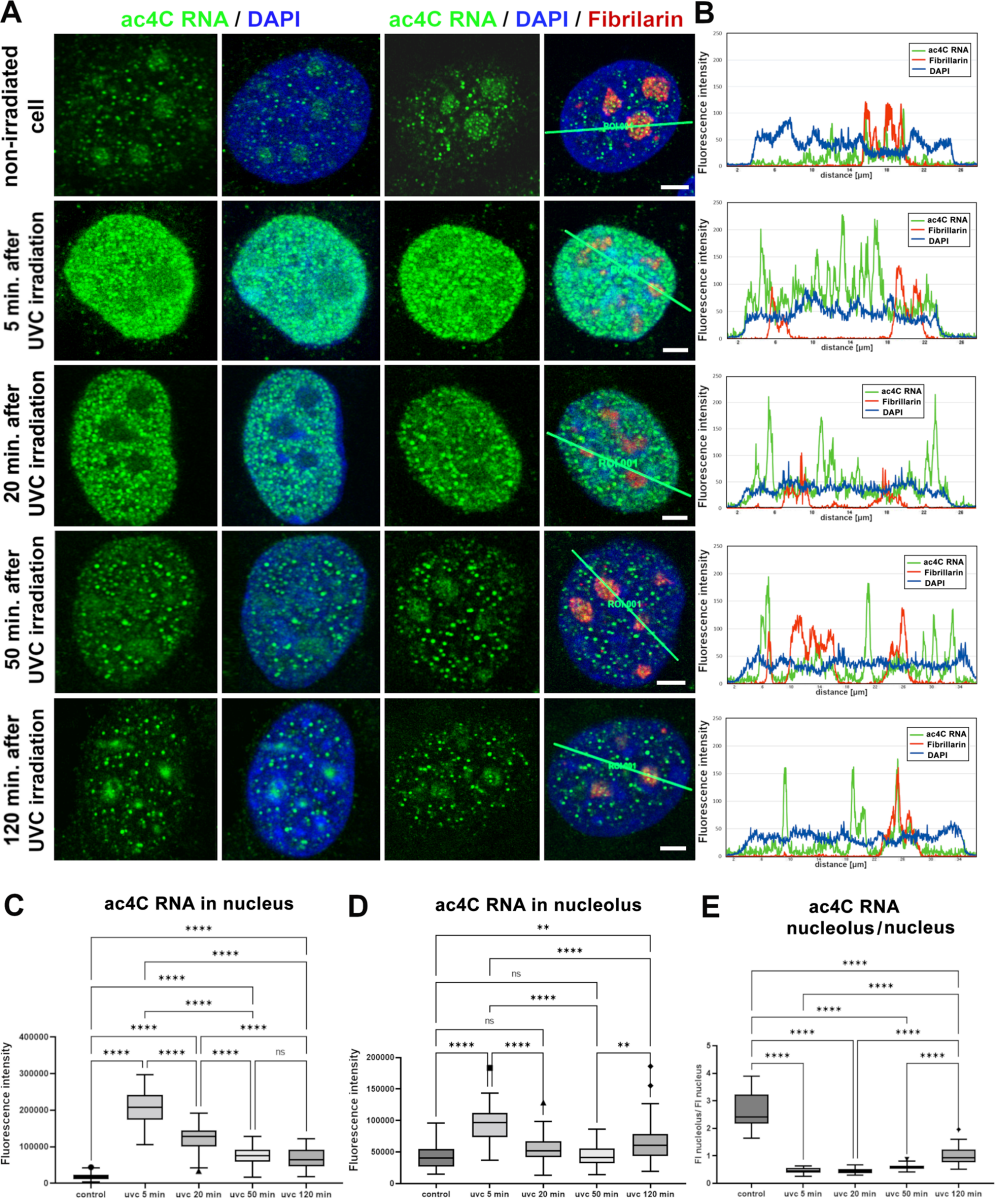
UVC light caused an accumulation of ac4C RNA into well-visible foci in a later stage of DDR. **A** In non-irradiated control cells, relatively high ac4C RNA positivity was observed in nucleoli (detected using immunostaining by the use of an antibody against fibrillarin). UVC irradiation increased the level of ac4C RNA in the whole nucleoplasm (the most marked changes were 5–20 min post-irradiation). MEFs analyzed 20–120 min after UVC irradiation were characterized by ac4C RNA reorganization into well-visible and ac4C RNA-dense tiny foci. Scale bars showed 5 µm. **B** Quantification shows the fluorescent intensity (FI) of ac4C RNA in the nucleoplasm (green) compared with fibrillarin-positive regions of nucleoli (red) and DAPI-stained DNA (blue). Quantification by LAS X software was performed across the green lines, shown in panel A. **C** Box plot graphs display the absolute intensity of ac4C RNA in the nucleoplasm (nucleus), ****p ≤ 0.0001 (ANOVA One-Way test). **D** Box plot graphs show the total intensity of fluorescently-stained ac4C RNA in nucleoli, ****p ≤ 0.0001, **p ≤ 0.01. **E** Box plot graphs depict the ratio of the fluorescent intensity of ac4C RNA occupying nucleoli and the nucleoplasm (whole nucleus), ****p < 0.0001

Notably, in comparison to the micro-irradiated genomic region, a pronounced level of ac4C RNA did not appear inside UVA-microirradiated nucleoli (the area labeled as Nu in Fig. 4B). However, a quantification of the density of ac4C RNA inside the nucleoli of UVC-irradiated cells showed that UVC-light slightly increased the level of ac4C RNA in nucleoli when compared to non-irradiated counterparts (Fig. 5A, B and D).

In these experiments, when the whole cell population was irradiated by a UVC lamp, we found a high density of ac4C RNA in the nucleoplasm 5—20 min post-irradiation. Cells analyzed 50–120 min after UVC-irradiation underwent ac4C RNA reorganization into well-visible, tiny foci (Fig. 5A). In UVC-irradiated cells, we quantified ac4C RNA distribution in the whole nucleus and the compartments of the nucleoli. As can be seen in Figs. 5A–E, ac4C RNA was concentrated in the nucleoli of non-irradiated cells. Conversely, after UVC irradiation, we observed higher fluorescence intensity (FI) in the nucleoplasm. The most marked changes in the nucleoplasm were detected 5 min after irradiation. In later intervals, 20 min after irradiation, the amount of ac4C RNA decreased in the entire cell nuclei. Statistically significant differences in absolute fluorescence intensity of ac4C RNA were observed within the cell nucleus when we compared non-irradiated and UVC-irradiated cells in distinct post-irradiation intervals (p ≤ 0.0001) (Fig. 5C). In comparison with control values, the total FI of ac4C RNA in the nucleoli was significantly higher in cells analyzed 5 min post-irradiation (Fig. 5D). The ratio of the FI in the nucleolus relative to the rest of ac4C RNA in the nucleus was 2.42 (median) in the control cells. For irradiated samples, at intervals of 5 min, 20 min, 50 min, and 120 min after irradiation, the FI range was from 0.44 to 0.94 (on average). The difference in relative FI was highly statistically significant in all irradiation intervals when compared with the control non-irradiated values (p ≤ 0.0001) (Fig. 5E).

### Accumulation of ac4C RNA in DNA lesions is PARP-dependent

**Fig. 6:**
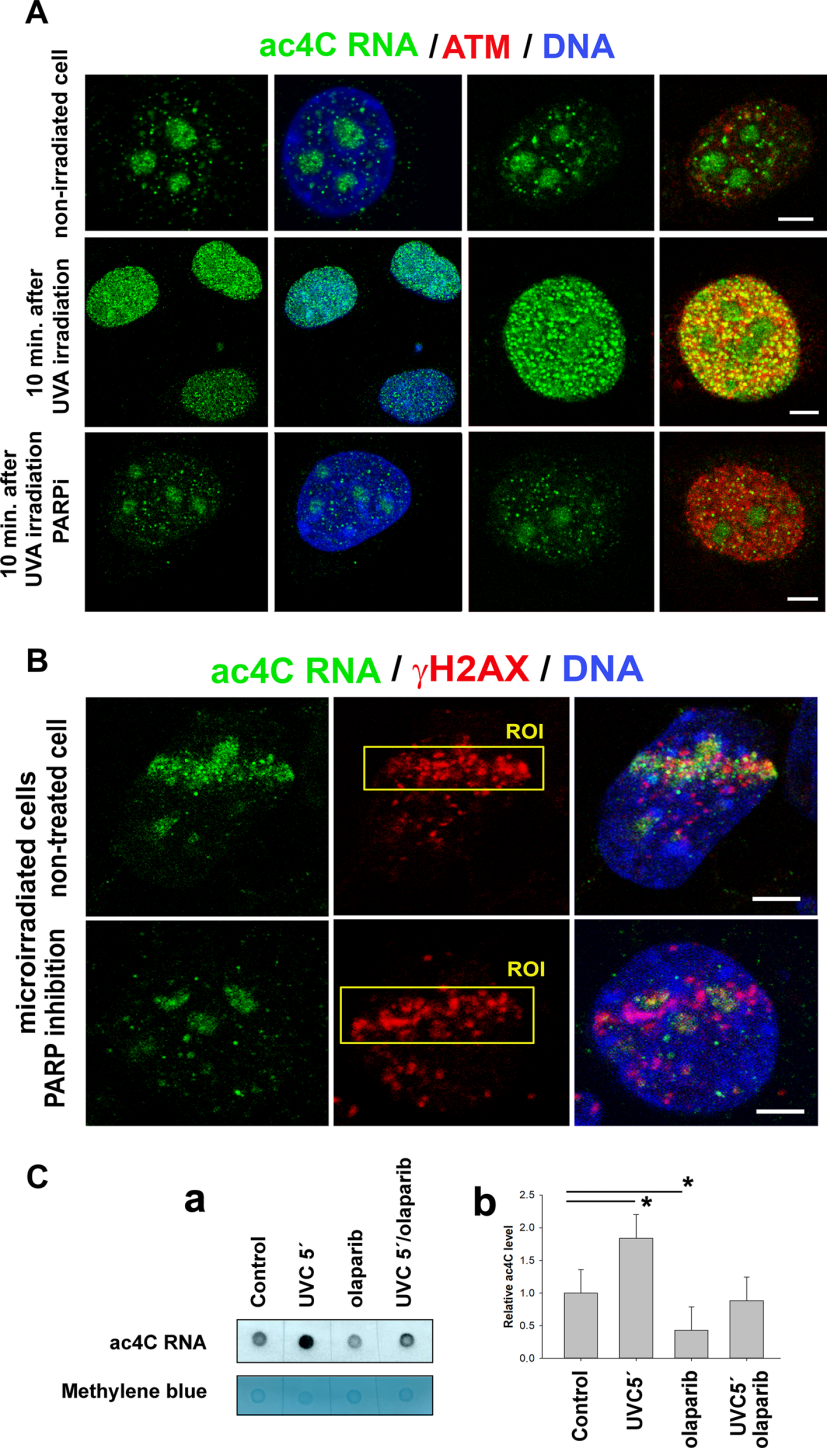
Recruitment of ac4C RNA to UVA-damaged chromatin is PARP-dependent. **A** UVA irradiated MEFs as a whole-cell population, and **B** microirradiated MEFs by the use of a 355-nm UVA laser. Local laser microirradiation showed that ac4C RNA did not accumulate to DNA lesions when the cells were treated with the PARP inhibitor. DNA damage was detected by antibodies against ATM and γH2AX. Scale bars are 5 µm. **C** anti-ac4C dot blot **a** (#ab252215, Abcam) in samples irradiated by UVC, treated by PARP inhibitor and treated with both PARP inhibitor and UVC irradiation. Panel **b** shows the quantification of the dot blot from (**a**)

We addressed the question of whether a pronounced appearance of ac4C at DNA lesions is PARP-dependent. We treated cells with a PARP inhibitor, olaparib (PARPi). In non-irradiated cells, we confirmed a high density of ac4C RNA in nucleoli, and 10 min after UVA irradiation, a high level of ac4C RNAs was detected in both nucleoli and the nucleoplasm (Fig. 6A). At the same time, cells treated by PARPi were characterized by the identical distribution profile of ac4C RNAs as was observed in control non-irradiated cells. The presence of DNA damage, induced by both UVA irradiation and PARPi, was evidenced by the high level of ATM (Fig. 6A). PARPi also prevents the recruitment of ac4C RNAs to UVA-microirradiated chromatin (Fig. 6B). Consistent with the IF results, dot blot using monoclonal antibody against ac4C demonstrated a near complete reduction of signal induced by UVC irradiation in total RNA (Fig. 6C, a, b). Thus, these data show that the accumulation of ac4C RNAs at DNA lesions is PARP-dependent (Fig. 6A–C).

### The NAT10 acetyltransferase is not recruited to UVA-damaged chromatin while the level of ac4C RNA is increased in the damaged genomic region

**Fig. 7:**
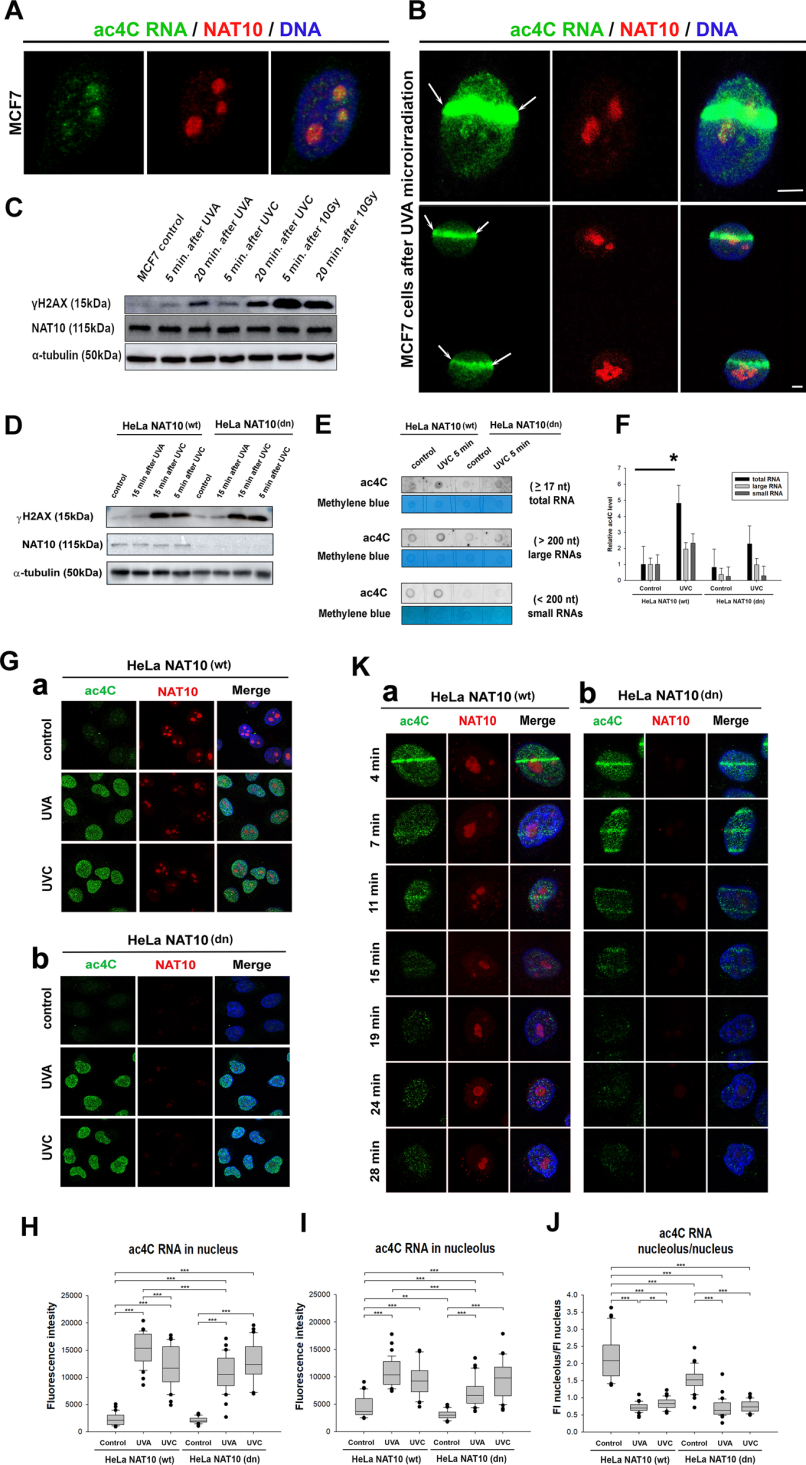
NAT10-independent recruitment of ac4C RNA to UVA-damaged chromatin. **A** Nuclear distribution of NAT10 (red) and ac4C RNA (green) in physiological conditions. MCF7 cells were studied instead of MEFs as antibodies are only available against the human epitope. **B** Local laser microirradiation showed that NAT10 acetyltransferase (red) does not recruit to DNA lesions (positive on ac4C RNA; green) induced in MCF7 cells. Scale bars are 5 µm. **C** Western blots showed no changes in NAT10 levels in cells exposed to UVA and UVC light. An effect of UV- as well as γ-radiation, was confirmed by an increased γH2AX level. **D** shows western blot results on the level of NAT10 and γH2AX in NAT10 (wt) and NAT10 (dn) cells. Western blot data were normalized to the level of α-tubulin, and proteins were loaded following the identical total protein levels. **E** A representative anti-ac4C dot blot (#A18806, Abclonal) was performed to study total, long, and small RNA in NAT10 (wt) and NAT10 (dn) cells. **F** Quantification of dot blot results from **E** is shown in panel F. Asterisks (*) indicate a statistically increased level of ac4C in RNA. **G** The level of ac4C RNA (green) and NAT10 (red) in non-irradiated control and UVA- or UVC-irradiated whole populations of **a** NAT10 (wt) and **b** NAT10 (dn) HeLa cells. **H** Box plot graphs display the absolute intensity of ac4C RNA in the nucleoplasm (nucleus), ***p ≤ 0.001 (ANOVA One-Way test). **I** Box plot graphs show the total intensity of fluorescently stained ac4C RNA in nucleoli, ***p ≤ 0.001, **p ≤ 0.01. **J** Box plot graphs depict the ratio of the fluorescent intensity of ac4C RNA occupying nucleoli and the nucleoplasm (whole nucleus), ***p < 0.001, **p ≤ 0.01. **K** The level of ac4C RNA (green) and NAT10 (red) in microirradiated **a** NAT10 (wt) and **b** NAT10 (dn) HeLa cells

It has been described previously that RNA cytidine acetyltransferase NAT10 (human acetyltransferase) is responsible for the installation of N4-acetyl cytidine (ac4C) on mRNAs, 18S rRNA, tRNAs, and lncRNA [2, 12, 13, 18]. Based on this information, we examined if the level of ac4C RNA at microirradiated chromatin is NAT10 dependent, even though NAT10 is prevalent in nucleoli and not the nucleoplasm. In this case, we found that NAT10 is not recruited to microirradiated genomic regions (Fig. 7A, B). It is known that knocking out NAT10 can significantly reduce ac4C levels [2]. Using western blot analysis, we compared the level of the NAT10 protein in wild-type and NAT10 double null cells [NAT10 (wt) and NAT10 (dn)] exposed to UVA and UVC light (Fig. 7C, D). Western blot results verified an absence of the NAT10 protein in NAT10 (dn) cells (Fig. 7D). However, we did not observe changes in NAT10 levels in wild-type cells exposed to UVA, UVC light, or γ-rays. In general, the radiation sources used increased the level of phosphorylated histone H2AX (γH2AX), a well-known epigenetic maker in the vicinity of double-strand breaks in DNA. However, the level of NAT10 remained stable (Fig. 7C, D). Also, using anti-ac4C dot blots, we analyzed total RNA, long and short RNAs isolated from wild-type (wt) and NAT10 (dn) HeLa cells. We found increased ac4C levels in total RNA, studied in (wt) cells irradiated by UVC light. As expected, we observed that cytidine acetylation is reduced in control non-irradiated NAT (dn) cells, but UVC light slightly increased the level of ac4C, especially in total RNA (Fig. 7E, F). Consistent with the results of dot blot analysis, immunohistochemistry demonstrated an identical increase in the level of ac4C RNA when UVA and UVC irradiated whole populations of NAT10 (wt) and NAT10 (dn) cells were compared to their non-irradiated counterparts (Fig. 7G–J). In comparison with the surrounding genome, the increased level of ac4C RNA was identical in microirradiated chromatin of NAT10 (wt) and NAT10 (dn) cells (Fig. 7K). Summarizing these data, it seems likely that ac4C accumulation in RNA at UV-induced DNA lesions is not mediated via the function of NAT10 acetyltransferase. These data imply the existence of another acetyltransferase responsible for the installation of ac4C in RNA.

### Recruitment of ac4C RNA to microirradiated chromatin was NHEJ independent

**Fig. 8:**
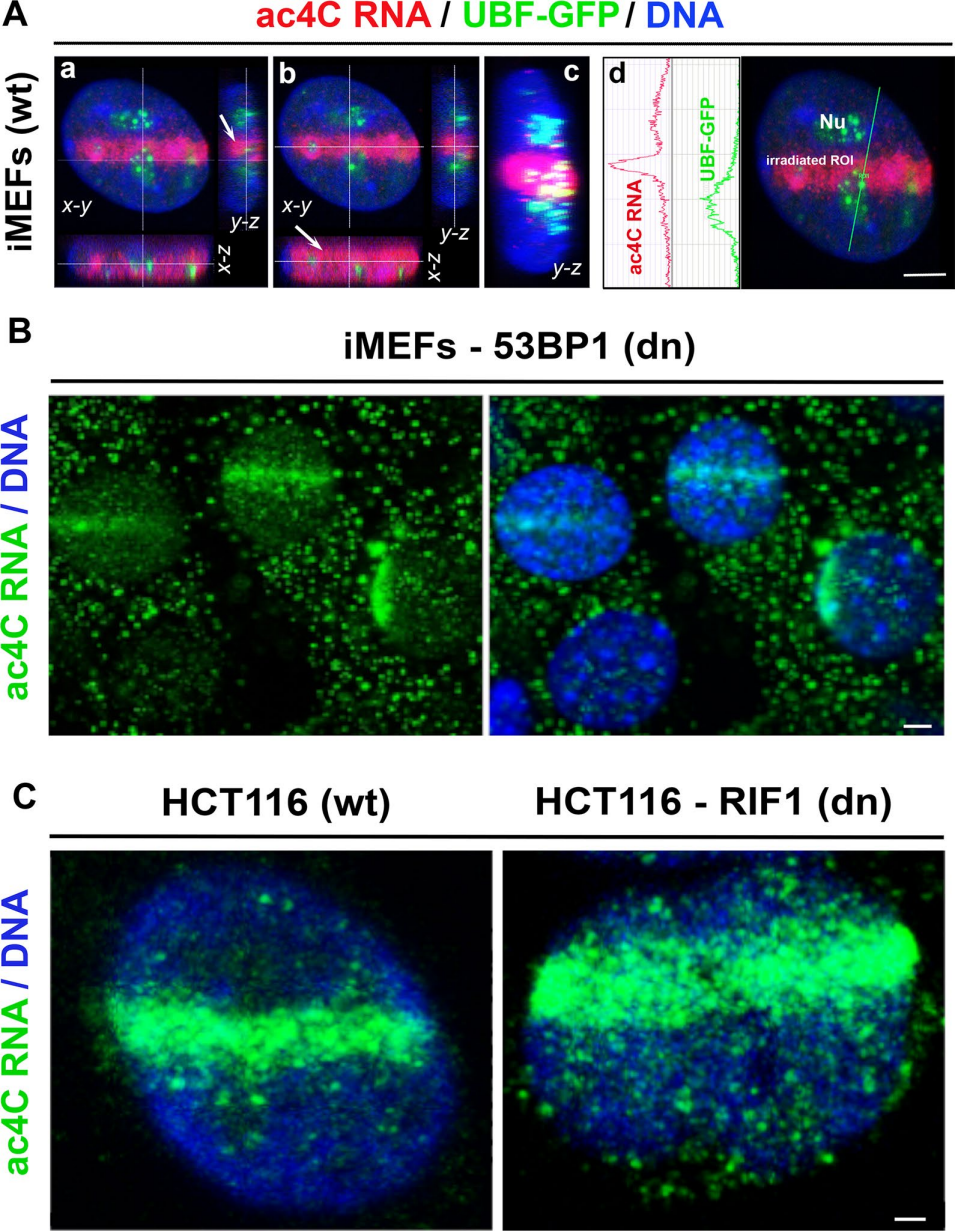
NHEJ independent recruitment of ac4C RNA to microirradiated chromatin. **A** 3D-projection of 60 confocal sections shows the high density of ac4C RNA (red) in microirradiated chromatin and in the proximity of nucleoli (visualized by GFP-tagged UBF1/2) [36]). Analysis was performed using immortalized wild type iMEFs. The 3D projection and density of ac4C RNA and GFP-UBFs were studied using LEICA AF software. **B** ac4C RNA was recruited to microirradiated chromatin in 53BP1 double null (dn) MEFs, in a similar density as shown in panel (A) for the wt MEFs. The scale bars show 5 µm. **C** ac4C RNA significantly accumulated at microirradiated chromatin of RIF1 (wt) and RIF1 double null (dn) HCT116 cells. The scale bar shows 2 µm

We next performed microirradiation analysis in cells with 53BP1 and RIF1 deficiency. Immunochemistry results showed that the increase in the level of ac4C RNA in microirradiated chromatin was identical when we compared wild type (wt) and 53BP1 double null (dn) or RIF1 (dn) cells (Fig. 8A–C). Using 3D-reconstruction of confocal sections, we additionally showed that microirradiation causes DNA damage in the whole nuclear content, as analyzed by z-projection (Fig. 8A). Moreover, we confirmed that irradiated nucleoli have a lower abundance of ac4C RNA than irradiated nucleoplasm (Fig. 4A, B and 8A).

Together, our results imply that increased ac4C RNA levels in microirradiated chromatin were not affected by a deficiency of the NHEJ repair factors that contribute to the repair of DSB sites, primarily in the G1 phase of the cell cycle. Conclusions from these experiments are additionally supported by the fact that acetylated RNAs recognize UV-induced DNA lesions not only in G1 (when NHEJ is activated) but also in S and G2 phases of the interphase when other DNA repair pathways, including HR, are engaged (Fig. 1A, B and [24]).

## Discussion

Acetylation processes and their regulation via specific epigenetic writers (acetyltransferases) and erasers (deacetylases) are well-described for histones. Another component of chromatin, DNA, lacks acetylation marks, but acetylation of N4-cytidine (ac4C) in RNA was revealed as a specific regulatory marker of the epitranscriptome [25]. From the view of nucleic acid biology and chromatin features, ac4C in RNAs is a unique biochemical epitranscriptomic modification. It is known that cytidine in RNA can be acetylated via a specific RNA acetyltransferase called NAT10, and ac4C in RNA can be erased via the function of sirtuin 7 [12, 13, 16]. Based on this knowledge and taking into account that other RNA modifications, including m^6^A RNA and m^8^A RNA, recognize UV-damaged chromatin [19], we investigated if there are changes in the level of ac4C RNA in UV-induced DNA lesions and if this process is NAT10-dependent. In this case, we observed a significant increase of ac4C RNAs at microirradiated chromatin of (wt) and NAT10 (dn) cells, but NAT10 did not recognize locally induced DNA lesions (Figs. 3A and 7A, B, K). This observation implies that in irradiated cells, a significantly increased level of ac4C RNA at damaged chromatin is NAT10-independent. Alternatively, there could be some NAT10-related co-factor mediating epitranscriptomic modification of RNAs, although, in our experimental model, NAT10 does not directly contribute to DNA damage repair. Another possibility is that another RNA acetyltransferase, responsible for ac4C installation on RNA, exists. Relevant to this, Xie et al. recently observed an enhanced ac4C RNA level in cells treated with cisplatin that also induces DNA damage. These authors suggested NAT10 as a therapeutic target to overcome cisplatin resistance in bladder cancers [26]. However, following our data, the role of NAT10 in DNA damage response, activated by anticancer therapy, must be elucidated.

It is well-known that RNA can also be methylated on N^6^-adenosine (m^6^A), which regulates many biological processes, including transcription, RNA stability, and translation [27]. From the perspective of DNA damage repair processes, Xiang et al. [19] showed that m^6^A RNA participates in DNA damage response. Conversely, the accumulation of m^6^A in DNA lesions was accompanied by a decrease of m^1^A and m_3_G/TMG [20]. Moreover, Zhang et al. [21] suggested the existence of a non-canonical and PARP-dependent DNA repair pathway mediated by the function of m^6^A RNAs that recognize UV-induced DNA lesions. Based on these results, we continued with our analysis of RNA modifications at DNA lesions, and we studied the pool of ac4C RNA in UV-damaged chromatin (Fig. 3A, B). As mentioned above, we observed high levels of ac4C RNA in microirradiated chromatin, and PARP inhibition reduced the level of ac4C RNA in the UVA-damaged genome (Fig. 6A, B). Thus, as with m^6^A RNA and m^8^A RNA, it would appear that the occurrence of ac4C RNA at microirradiated chromatin is PARP dependent ([19, 27] and Fig. 6A, B). Also, we confirmed by dot blots that ac4C is reduced in samples treated by PARP inhibitor, and combinatory treatment by both PARP inhibitor and UVC irradiation diminished the effect of UVC on the elevation of ac4C in RNA (Fig. 6C, a, b).

A very important observation is that ac4C RNA, compared to m^6^A RNAs and m^8^A RNA, appears at DNA lesions over a different time interval (Fig. 3A and [20, 27]). In damaged chromatin, a high density of ac4C RNA is present 2–45 min post-irradiation while both m^6^A RNA and m^8^A RNA signals appear in the genome immediately after microirradiation, where levels remain stable up to 5 min ([20, 27]; Fig. 3A). These observations show that there are different kinetics and functions of ac4C RNA and methylated RNAs in UV-damaged chromatin. It is probable that ac4C RNA contributes to chromatin de-condensation in the later stages of the DNA damage response, which is likely not simply the case of methylated RNAs occupying DNA lesions for a very short interval after UV light exposure. Regarding the later DNA damage response, our previous experiments showed that BRCA1 is recruited to DSB-sites 20 min post-irradiation, which fits well with the kinetics of ac4C RNA at UV-damaged chromatin ([27]; Fig. 3A). Identical kinetics of BRCA1 and ac4C RNA could imply the function of acetylated RNAs in the homologous recombination (HR) repair pathway that proceeds in S/G2 phases of the cell cycle. However, we observed ac4C RNA signals also in the G1 phase of the cell cycle when the NHEJ repair pathway is preferentially activated if we are discussing the repair of DSBs (Fig. 1A, B). Based on these results, we studied the localized kinetics of ac4C RNA in 53BP1 (dn) and RIF (dn) cells, and we showed that depletion of both NHEJ-related factors did not affect the accumulation of ac4C RNA at UV-damaged genome (Fig. 8A, B). Our data instead likely support the existence of a non-canonical m^6^A/m^8^A/ac^4^C-mediated DNA repair pathway dependent on PARP function. Alternatively, modified RNAs could be involved in the BER pathway, recognizing single-strand damage in DNA. To this fact, recently, we observed PARP-dependent recruitment kinetics of both m^8^A RNA and the XRCC1 protein [21, 27]. Another possibility, in this case, is that RNA modifications, including m^6^A, m^8^A, and ac4C, could be simply markers of UV-damaged RNA.

Taken together, we suggest that modified RNAs, preferentially small RNAs (Fig. 3B, C), significantly contribute to DNA damage repair, and this process is PARP-dependent but independent of the function of RNA acetyltransferase NAT10. We suggest that the role of methylated RNAs could be linked to the stabilization of DNA lesions immediately after irradiation, while in a later step of DDR, ac4C RNA likely maintains chromatin de-condensation, which is essential for active DNA demethylation that appears at UV-damaged chromatin ([20, 27]]; Fig. 3A). This observation documents that the DNA repair machinery is characterized by changes not only in histone signature but also modifications of nucleic acids.

## Materials and methods

### Cell cultivation and treatment

Mouse embryonic fibroblasts (MEFs) and human breast cancer cell line MCF7 were maintained in Dulbecco's modified Eagle's medium (DMEM; Merck, Darmstadt, Germany) supplemented with 10% fetal bovine serum FCS (Merck), penicillin (1 U/ml), and streptomycin (100 μg/ml) at 37 °C in a humidified atmosphere containing 5% CO_2_. For dependent recruitment of ac4C on cell cycle experiments, we used HeLa-Fucci cells expressing RFP-Cdt1 in the G1 phase and GFP-geminin in the S/G2/M phases, as have previously been described in detail by Sakaue-Sawano et al. [23]. HeLa-Fucci cells were cultivated in the same in vitro conditions. 53BP1-deficient and 53BP1 wild-type immortalized mouse embryonic fibroblasts (iMEFs) were a gift from Michela Di Virgilio, Laboratory of DNA Repair and Maintenance of Genome Stability, Max Delbrück Center for Molecular Medicine in the Helmholtz Association, Berlin, Germany. Immortalized MEFs were cultured in DMEM medium supplemented with 10% FBS, 2 mM L-Glutamine, and Penicillin–Streptomycin at 37 °C and 5% CO_2_ [28]. RIF1-deficient HCT116 cells and wild-type HCT116 cells (a generous gift from Prof. David M. Gilbert, San Diego Biomedical Research Institute, USA, [29]) were cultivated in DMEM (Merck) supplemented with 10% FBS, and Penicillin–Streptomycin. Wild-type (wt) and NAT10 double null (dn) HeLa cells were cultured in DMEM and supplemented with 2 mM L-glutamine, 10% bovine calf serum without antibiotics, and maintained in a thermostat at 37 °C, supplemented with 5% CO_2_. These cells were a generous gift from Dr. Shalini Oberdoerffer, NCI NIH, USA (Arango et al. [2, 17]).

We inhibited RNA polymerase I or poly (ADP ribose) polymerase (PARP). In this case, cells were treated at 50% confluence with actinomycin D (#A9415, Merck; final concentration 0.5 µg/ml, 2 h treatment before microirradiation), or by olaparib (#S1060, Selleckchem, Germany; final concentration 10 µM, treatment 24 h before microirradiation) [30, 31].

Treatments by enzymes: We used Turbo-DNase (#AM2238, Thermo Fisher Scientific, Waltham, MA, USA), RNase A (#R5503, Merck), and RNase H1 (#EN0201, Thermo Fisher Scientific). The cells were permeabilized with cold 0.1% Triton X-100 in PBS for 10 s, washed twice in phosphate-buffered saline (PBS), and incubated in 300 μl RNase A (0,5 mg/ml in PBS) or DNase I (5 U in 1 × DNase Reaction buffer) or RNase H1 (2U in 1 × RNase Reaction buffer) for 8 min at 37 °C before immunostaining [19, 32]. Subsequently, fixation was performed with 4% formaldehyde and permeabilization with 0.3% Triton X. After that, it was followed by further enzymatic treatment for 1 h at 37 °C. Relative fluorescence intensity was evaluated in 25 nuclei, and statistical analysis was performed. For dot blot analysis, digestion was performed by RNase A (2U/5 µg RNA), RNase H1 (2U/5 µg RNA), and DNase I (0.8 U/5 µg RNA) and incubated for 15 min at 37 °C.

### Irradiation by UV-light

Cells seeded on 35 mm glass-bottom dishes (#D35-20-1-N, Cellvis Mountain View, CA, USA) and at 50% confluence were sensitized with 10 μM BrdU (#11296736001, Merck) for 16 h before UVA treatment. Cells were irradiated by the UVA lamp (model GESP-15, 15 W, UVA 330–400 nm wavelength, with maximum efficiency at 365 nm) or UVC lamp (Philips, Amsterdam, The Netherlands, model TUV 30 W T8, UVC 254 nm wavelength). Irradiation was performed for 10 min. After UVC irradiation, the cells were fixed at multiple intervals (5 min, 20 min, 60 min, and 120 min after irradiation). The lamp distance from the sample was 2 cm for the UVA source and 60 cm for the UVC source [20]. Statistical analysis was performed for 40 cells.

### Immunofluorescence and confocal microscopy

The immunofluorescence protocol was adapted according to Svobodova Kovaříková et al. [33] and modified. Cells were fixed with 2 ml 4% formaldehyde (prepared from paraformaldehyde, PFA; #AAJ19943K2, Thermo Fisher Scientific) for 5 min at room temperature (RT), and then 200 ml 1% SDS was added, and initial incubation was extended by an additional 7 min. Afterward, samples were permeabilized with 0.2% Triton X-100 for 15 min and washed twice in PBS for 15 min. As a blocking solution, we used 1% bovine serum albumin (Merck), dissolved in 0.1% 1 × PBS-Tween 20 (BSAT) for 1 h at RT Dishes with fixed cells were washed for 15 min in PBS and incubated with primary antibodies at a 1:100 dilution in 1% BSAT at 4 °C overnight. For immunofluorescence analysis, the following antibodies were used: anti-N4-acetylcytidine/ac4C in RNA (#A18806 Abclonal, Woburn, MA, USA), anti-phosphorylated histone H2AX (γH2AX; phospho S139) (#05-636, Merck), anti-fibrillarin (#ab4566, Abcam), anti- phospho-ATM; Ser1981 (#MAB3806-C, Merck), anti-α-tubulin (#ab80779 Abcam), and anti-NAT10 (B-4) (#sc-271770, Santa Cruz Biotechnology, Dallas, TX, USA). After incubation with primary antibodies, the samples were washed twice in PBS for 15 min and incubated with the following secondary antibodies, diluted 1:300 in 1% BSAT: Alexa 488-conjugated goat anti-mouse (#ab150077, Abcam), Alexa 594-conjugated goat anti-rabbit (#A11037, Thermo Fisher Scientific), Alexa 488-conjugated goat anti-mouse (#A11029, Thermo Fisher Scientific), Alexa Fluor 594-conjugated goat anti-mouse (#A11032, Thermo Fisher Scientific), and Alexa 647-conjugate goat anti-rabbit (#A21245, Thermo Fisher Scientific). The DNA content was visualized using 4′,6-diamidino-2-phenylindole (DAPI; Merck), and Vectashield (Vector Laboratories, USA) was used as the mounting medium. Samples were also incubated without primary antibodies for negative control staining.

### Local laser microirradiation and laser scanning confocal microscopy

For the microirradiation experiments using UVA lasers (wavelength 355 nm), cells were seeded on 35 mm gridded microscope dishes (#81,166, Ibidi, Fitchburg, WI, USA), and at 50% confluence, cells were sensitized with 10 μM BrdU for 16 h. For microscopy, the cells were maintained under optimal cultivation conditions in an incubation chamber (EMBL) at 37 °C, supplemented with 5% CO_2_. In the selected cell nuclei, we irradiated only the defined region of interest (ROI) using a laser connected to TCS SP5-X confocal microscope system (Leica, Wetzlar, Germany). The microscope settings for induction of local DNA damage were as follows: laser power (355 nm) 25 mW, 512 × 512 pixel resolution, 400 Hz, bidirectional mode, 48 lines, zoom 4, and 63 × oil objective (HCX PL APO, lambda blue) with a numerical aperture (NA) = 1.4 [20]. The maximum exposure of the cells to the laser was 45 min, and we monitored approximately 100 cell nuclei. Analysis of 3 biological replicates was performed. After the immunostaining procedure, locally microirradiated cells were localized according to registered coordinates on gridded microscope dishes. We studied the level of the epigenetic marker N4-acetylcytidine in RNA, NAT10 acetyltransferase, and the presence of γH2AX (phospho S139), which was also used for the optimization of microirradiation experiments. For image acquisition and analysis of fluorescence intensity (FI) we used LEICA LAS X software.

### Western blotting

Western blotting was performed using the methods reported in [33]. We used the following primary antibodies: anti-phosphorylated histone H2AX (γH2AX; phospho S139; #ab2893, Abcam), anti-α-tubulin (#ab80779 Abcam), anti-NAT10 (B-4) (#sc-271770, Santa Cruz Biotechnology), and antibody against α-tubulin (#ab80779 Abcam). As secondary antibodies, we used anti-rabbit IgG (#A-4914, Merck; dilution 1:2000), anti-mouse IgG (#A-9044, Merck; dilution 1:2000) and anti-mouse IgG1 (#ab97240, Abcam; dilution 1:5000).

### Isolation of total, long and small RNA

Total RNA was purified from MEFs (the second day after seeding) using the Quick-RNA Miniprep Kit (#R1054; Zymo Research, Irvine, CA, USA). For isolation of small RNA was used mirVana™ miRNA Isolation Kit (#AM1560, Thermo Fisher Scientific). RNA isolations were done according to the manufacturer's instructions. Large forms of RNA were separated using both kits in a special step according to the manufacturer's instructions. Following purification, RNA was quantified using a spectrophotometer (NanoDrop™ 2000/2000c Spectrophotometers (#ND-2000, Thermo Fisher Scientific). RNA samples for Dot blot analysis and mass spectrometry were isolated from 3 biological replicates.

### Gel analysis of RNA

RNAs (1–1,5 µg) intended for dot blotting were mixed with 2 × RNA loading dye (#R0641, Thermo Fisher Scientific) in a ratio of 1:1 and heated to 70 °C for 5 min, followed by chilling on ice. These RNA samples were separated in a 10% denaturing polyacrylamide gel containing 7 M urea. As running buffer was used 1 × TBE (90 mM Tris, 90 mM borate, and 2 mM EDTA, pH = 8.0) and electrophoresis conditions were 35 mA for 1 h in the cold. After electrophoretic separation, the gel was washed in 1 × TBE for 10 min and incubated in GelRed (#41,003, Biotium, Fremont, CA, USA) for 15 min. Additional washing was in 1 × TBE. The RNA bands were visualized using an Amersham Imager 680 (GE Healthcare, Freiburg, Germany).

### Dot blots

Samples of RNA were diluted to a final concentration of 250 ng/µl or 200 ng/µl. The method was based on the Abcam RNA Dot Blot Protocol (https://www.abcam.com/protocols/rna-dot-blot-protocol) and modified according to our conditions. Diluted RNA was denatured at 95 °C in a heat block for 3 min, immediately placed on ice for 1 min, and loaded onto Hybond-N + membranes. Membranes were crosslinked by UVC 254 nm lamp for 30 min. The parameters were calculated so that the total energy was 300 mJ/cm2. After that, membranes were washed in 10 ml of TBST (1X TBS, 0.1% Tween-20) for 5 min at RT, blocked with 4% non-fat milk in TBST for 1 h at RT with gentle shaking, and incubated overnight with primary antibodies anti-N4-acetylcytidine/ac4C (#ab252215, Abcam or #A18806, Abclonal) in blocking buffer, at 4 °C (dilution 1:2000 or 1:1000). Membranes were washed three times for 10 min in TBST. As the secondary antibody, we used anti-rabbit IgG (#A-4914, Merck; dilution 1:5000) and visualized spots by Amersham Imager 680 (GE Healthcare). The loading was determined by 0.02% methylene blue stain (R.0648.1, P-LAB, Czech Republic). Chemical deacetylation was induced by hydroxylamine (50 mM, pH = 7, 65 °C, 1 h), according to [22]*.*

### Mass spectrometry

Analysis by mass spectrometry we performed in the core facility of the Central European Institute of Technology (CEITEC) in Brno. Isolation of RNA was performed as described above, and standard N4-acetylcytidine, for mass spectrometric measurement of the ac4C level in RNA, was purchased from BIZOL company, cat. number #CBS-NA05753; CAS [3736-18-1], Biosynth; https://www.biozol.de/en/product?q=CBS-NA05753. RNA modification analysis protocol was adapted from S. Kellner et al. [34, 35]. Shortly, RNA (10 µg) was digested with 0.1U P1 nuclease, 0.3U Snake venom phosphodiesterase, and 20 ng/µl pentostatin for 2 h at 37 °C. Dephosphorylation was performed using Shrimp alkaline phosphatase in 1 × Phosphatase buffer (we added 1/10 volume of 100 mM MgCl_2_, 100 mM ammonium acetate, pH 9.0) for 1 h at 37 °C and filtered through Microcon-10 filters (Merck). The nucleosides were separated on a YMC-Triart C18 column (100 × 3.0 mm ID, S-3 µm, 12 nm, YMC) and analyzed using HPLC Agilent 1260 Infinity system (Agilent) (Additional file 2: Table S1). The canonical nucleoside level was measured using a 1260 infinity DAD detector and quantified using an Agilent 6460 Triple Quad Mass Spectrometer.

### Statistical analysis

Fluorescence intensity values were measured by LAS X software and subsequently analyzed in Python 3 software. The obtained data were compared statistically using the ANOVA One-Way test, available in GraphPad Prism software, version 9 for Windows (GraphPad Software, San Diego, CA, USA). Also, the Student's t-test (Sigma Plot software, version 14.5; Systat Software, Inc., USA) was used for statistical analysis. All values labeled in the graphs by the asterisk(s) differ significantly from the control values.

## Supplementary Information

**Additional file 1: Fig. S1.** Ac4C level after enzymatic treatment. **A** Cells were treated by RNase A, RNase H1, and DNase I to study the level of ac4C RNA (anti-ac4C, #A18806, Abclonal) and the density of DNA in MCF7 cells. After RNase A treatment, the fluorescent intensity of ac4C RNA was significantly reduced, which was not the case in the following RNase H1 and DNase I treatments. Significant changes in the level of DAPI-stained DNA were observed when cells were treated with DNase I. Quantification of fluorescence intensities from panel A is shown in panels **B**, **C**. **D** Dot blot analysis of an ac4C RNA after enzymatic treatment using both ac4C antibodies (#ab252215, Abcam, or #A18806, Abclonal). Representative anti-ac4C dot blot was performed on total RNA with methylene blue as a loading control. **E** Dot blot analysis demonstrates chemical deacetylation by hydroxylamine (50 mM, pH 7.0, 65 °C, 1 h) detected by both antibodies. **F** Agarose gel shows fractionalization of RNA into large and small RNAs.

**Additional file 2:Table S1.** HPLC gradient timetable and source and acquisition parameters.
