# Supplementary figures and images for: PARP-dependent and NAT10-independent acetylation of N4-cytidine in RNA appears in UV-damaged chromatin
Source: Epigenetics Chromatin. 2023 Jun 15;16:26. doi: 10.1186/s13072-023-00501-x (PMC10268562; doi:10.1186/s13072-023-00501-x)

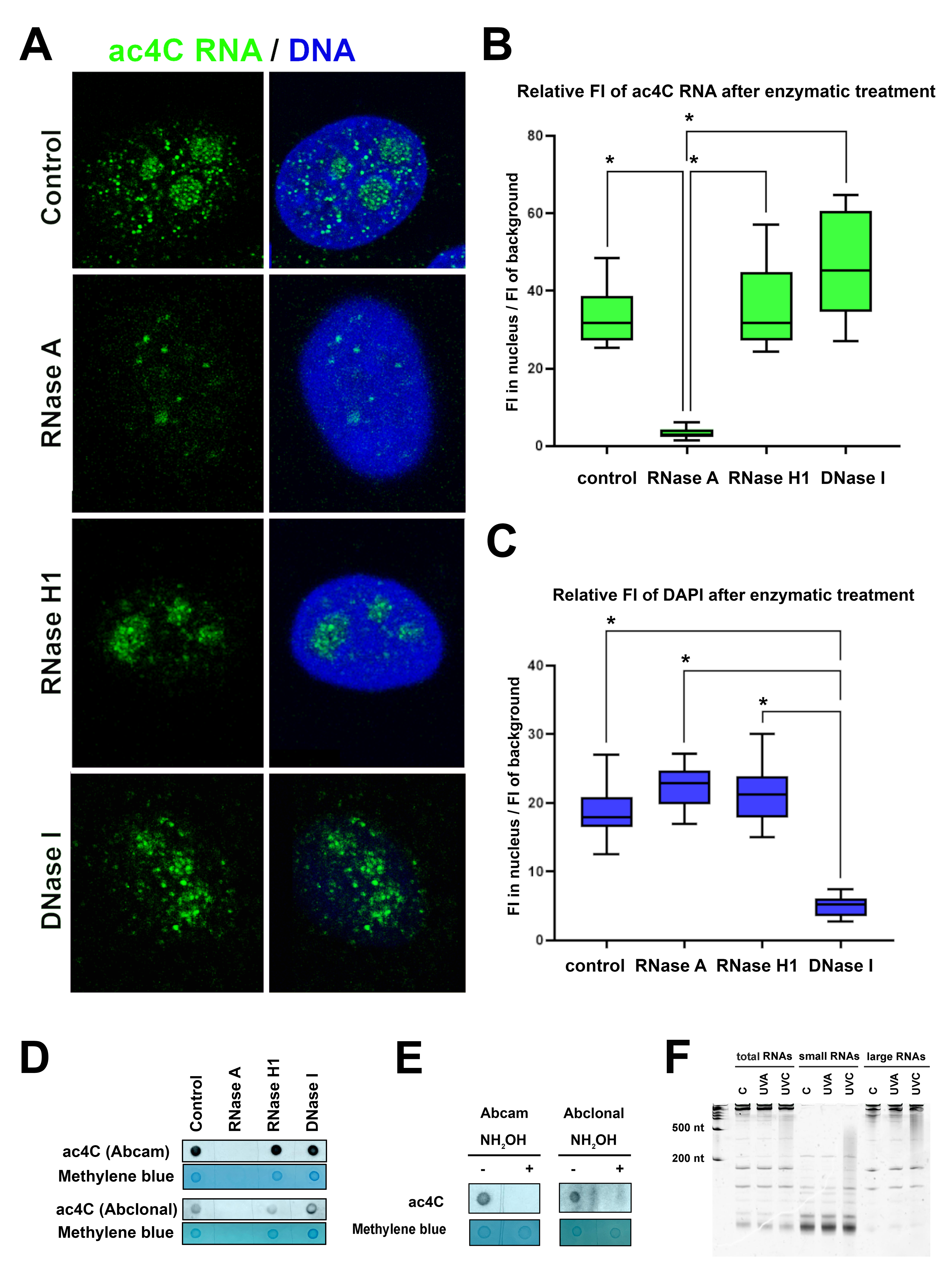

Supplement: Supplementary file 1 — Additional file 1: Fig. S1. Ac4C level after enzymatic treatment. A Cells were treated by RNase A, RNase H1, and DNase I to study the level of ac4C RNA (anti-ac4C, #A18806, Abclonal) and the density of DNA in MCF7 cells. After RNase A treatment, the fluorescent intensity of ac4C RNA was significantly reduced, which was not the case in the following RNase H1 and DNase I treatments. Significant changes in the level of DAPI-stained DNA were observed when cells were treated with DNase I. Quantification of fluorescence intensities from panel A is shown in panels B, C. D Dot blot analysis of an ac4C RNA after enzymatic treatment using both ac4C antibodies (#ab252215, Abcam, or #A18806, Abclonal). Representative anti-ac4C dot blot was performed on total RNA with methylene blue as a loading control. E Dot blot analysis demonstrates chemical deacetylation by hydroxylamine (50 mM, pH 7.0, 65 °C, 1 h) detected by both antibodies. F Agarose gel shows fractionalization of RNA into large and small RNAs. [file 13072_2023_501_MOESM1_ESM.tif]
